# Tumor Microenvironment: A Complex Landscape of Cancer Development and Drug Resistance

**DOI:** 10.7759/cureus.82090

**Published:** 2025-04-11

**Authors:** Sohaila Fatima

**Affiliations:** 1 Pathology, College of Medicine, King Khalid University, Abha, SAU

**Keywords:** cancer development, drug resistance, immunotherapy, targeted medicines, tumor microenvironment

## Abstract

Cancer is responsible for nearly one in six global fatalities, making it a major health issue worldwide. Despite advancements in early detection, surgery, and targeted therapies, effective treatment remains challenging due to the complexity and heterogeneity of the disease. A key factor in cancer progression and resistance to treatment is the tumor microenvironment (TME). It is a complex ecosystem comprising cancer cells, stromal cells, immune cells, extracellular matrix (ECM), and soluble factors like cytokines and chemokines. These components interact dynamically to influence tumor growth, metastasis, immune evasion, and treatment resistance. Cancer cells drive the formation of the TME by releasing signaling molecules, while stromal cells, such as fibroblasts and endothelial cells, support tumor metabolism, angiogenesis, and invasion. Immune cells within the TME can either suppress or promote tumor progression, depending on their activation state. Additionally, the TME can promote the growth of immunosuppressive cells that aid cancer cells in evading immune surveillance, such as regulatory T-cells and myeloid-derived suppressor cells. The TME also impedes drug delivery by creating defective blood vessels, contributing to drug resistance. Recent technological advancements have deepened our understanding of the TME, revealing its role in immune modulation, metabolism, and extracellular matrix remodeling. As a result, targeting the TME has become a promising strategy to overcome treatment resistance and improve cancer therapy outcomes.

## Introduction and background

Almost one in six fatalities globally are caused by cancer, making it one of the biggest global health issues. Cancer continues to be a major cause of death, with an anticipated 19 million new cases and almost 10 million deaths from the disease in 2020 alone [1]. Effective treatment of cancer is still hampered by its complexity and heterogeneity, even with significant improvements in early detection, surgical procedures, targeted medicines, and immunotherapies. The tumor microenvironment (TME) plays a crucial role in the development of cancer and treatment resistance [2].

Cancer cells are surrounded by a complex and dynamic ecosystem known as the TME, which is made up of both cellular and non-cellular elements that work together to affect tumor growth, metastasis, and treatment outcomes [3]. The TME comprises a range of stromal cells, immune cells, blood vessels, extracellular matrix (ECM) components, and soluble substances, such as growth factors and cytokines, in addition to malignant cells [4]. These components interact in ways that can either promote or prevent the growth of tumors. Cancer cell activity, including their capacity to multiply, infiltrate nearby tissues, elude immune surveillance, and eventually spread to distant organs, is greatly influenced by the TME [5]. It is well recognized that the TME offers tumor cells a favorable environment that encourages their growth, survival, and metastasis. It has a key mediating role in the development of the malignant phenotype [6]. The TME's capacity to modify immune system function, frequently by attracting immunosuppressive cells such as regulatory T-cells (Tregs) and myeloid-derived suppressor cells (MDSCs), is one of its primary characteristics [7]. These cells aid tumor cells in avoiding immune surveillance, which permits malignancies to proliferate unchecked. The TME uses acquired or de novo pathways to cause chemotherapeutic resistance. Cellular crosstalk and cell-to-TME-matrix contact are the means by which oncogene activation, tumor-suppressor gene dysregulation, and the production of ATP-binding cassettes (ABCs) are accomplished in acquired multidrug resistance (MDR). Cancer cells that have been exposed before have phenotypic alterations that make them resistant to further treatment [8]. However, in de novo resistance, the stromal tissue in the TME harbors a subset of cancer cells and, by promoting stemness, makes them resistant to chemotherapy following therapeutic exposure [9]. In the TME, tumor cells can adjust to extreme conditions such as low oxygen (hypoxia), acidic pH, and nutritional restriction [10]. These modifications may increase resistance to standard treatments like radiation, chemotherapy, and targeted therapies. Therefore, knowing how the TME affects therapeutic resistance is essential for creating plans to get beyond these obstacles and enhance patient outcomes [11].

Understanding the relationships between tumor cells and the TME has gained attention recently in an effort to find possible treatment targets. Technological developments like imaging, spatial transcriptomics, and single-cell ribonucleic acid (RNA) sequencing have shed light on the intricate relationships that control tumor behavior and the diverse nature of the TME [12,13]. The significance of the tumor vasculature, immune cell infiltration, metabolic reprogramming, and extracellular matrix remodeling in forming the tumor microenvironment has been demonstrated by these investigations [14,15].

This review will explore the role of the TME in cancer progression, highlighting its influence on tumor biology, immune evasion, drug resistance, and its potential as a therapeutic target. The review will also examine emerging strategies aimed at targeting the TME to enhance the efficacy of cancer therapies.

## Review

Methodology

A comprehensive search was performed across multiple scientific databases, including PubMed, Scopus, and Web of Science. The search utilized a combination of keywords such as tumor microenvironment, cancer development, drug resistance, immune modulation, and extracellular matrix. The search was limited to studies published in English and those involving human subjects.

*Selection Criteria* 

Inclusion criteria: Studies that investigated the role of the TME in cancer progression, metastasis, and drug resistance; studies that focused on the cellular or molecular mechanisms underlying TME-mediated immune evasion or immune modulation; and studies that explored therapeutic strategies targeting the TME.

Exclusion criteria: Studies that were unrelated to the topic, those focusing on animal models only, and articles that lacked full-text access or detailed experimental data.

Cancer cells in the TME

Role in Tumor Initiation and Progression

Cancer cells are the basic building block of all tumors and are essential to their development and spread [6]. When a single cell experiences genetic or epigenetic changes that enable it to evade typical growth regulators like apoptosis and senescence, a tumor is initiated [16]. This change often comes on by mutations in tumor suppressor genes or proto-oncogenes, which cause unchecked cell division [17]. In addition to spreading locally, cancer cells also affect the surrounding TME as the tumor grows by releasing substances that support immune evasion, angiogenesis (the formation of new blood vessels), and extracellular matrix modification [18].

Genetic and Epigenetic Alterations in Tumor Cells

The malignant behavior of tumor cells is influenced by genetic and epigenetic alterations. Mutations, deletions, or amplifications of particular genes are examples of genetic changes that can cause uncontrolled cell division and survival. For instance, a variety of malignancies frequently exhibit mutations in tumor suppressor genes (like tumor protein p53 (or TP53) or breast cancer type 1 BRCA1) or oncogenes (like Kirsten rat sarcoma virus (KRAS) or epidermal growth factor receptor (EGFR)) [19]. Dysregulated cell cycle regulation, abnormal signaling pathways, or resistance to cell death processes could result from these alterations [20]. By changing patterns of gene expression without changing the underlying DNA sequence, epigenetic modifications such as deoxyribonucleic acid (DNA) methylation, histone modifications, and non-coding RNA changes can also aid in the development of tumors [21]. These epigenetic changes may cause oncogenes to become active or tumor suppressor genes to be silenced, which would accelerate the growth of cancer and make it more resistant to treatment [22]. Cancer cells can adapt to the environmental forces seen in the TME, including hypoxia, food restriction, and immune surveillance, through both genetic and epigenetic changes. This leads to tumor growth and resistance to treatment [15].

Concept of Tumor Heterogeneity Within the TME

The existence of various populations of cancer cells within a single tumor is referred to as tumor heterogeneity [23]. A pool of cells with differing capacities to proliferate, invade, and withstand treatment can result from this heterogeneity, which can be caused by genetic mutations, epigenetic modifications, and different reactions to the TME [24]. Two types of tumor heterogeneity can be distinguished: inter-tumor and intratumor heterogeneity.

Inter-tumor heterogeneity: Variations between patient tumors, even within the same cancer type. Personalized treatment techniques are essential since these variations can impact prognosis and therapeutic response.

Intra-tumor heterogeneity: Differences in the genetic, epigenetic, and phenotypic traits of cancer cells inside the same tumor. As the tumor grows, subpopulations of cancer cells acquire unique mutations that provide them a competitive advantage, leading to intra-tumor heterogeneity. This is caused by clonal evolution. As the tumor grows, subpopulations of cancer cells acquire unique mutations that provide them a competitive advantage, leading to intra-tumor heterogeneity. This is caused by clonal evolution. Tumor cell interactions with their surrounding TME further contribute to this heterogeneity [25].

The heterogeneity of cancer cells in the TME makes treatment plans more difficult because various subpopulations may react differently to the same treatment. Relapse and treatment failure may result from sub-clones that are more resistant to immunotherapy or chemotherapy [26]. Developing successful treatment plans and overcoming resistance mechanisms require an understanding of and attention to tumor heterogeneity within the TME. 

**Figure 1:**
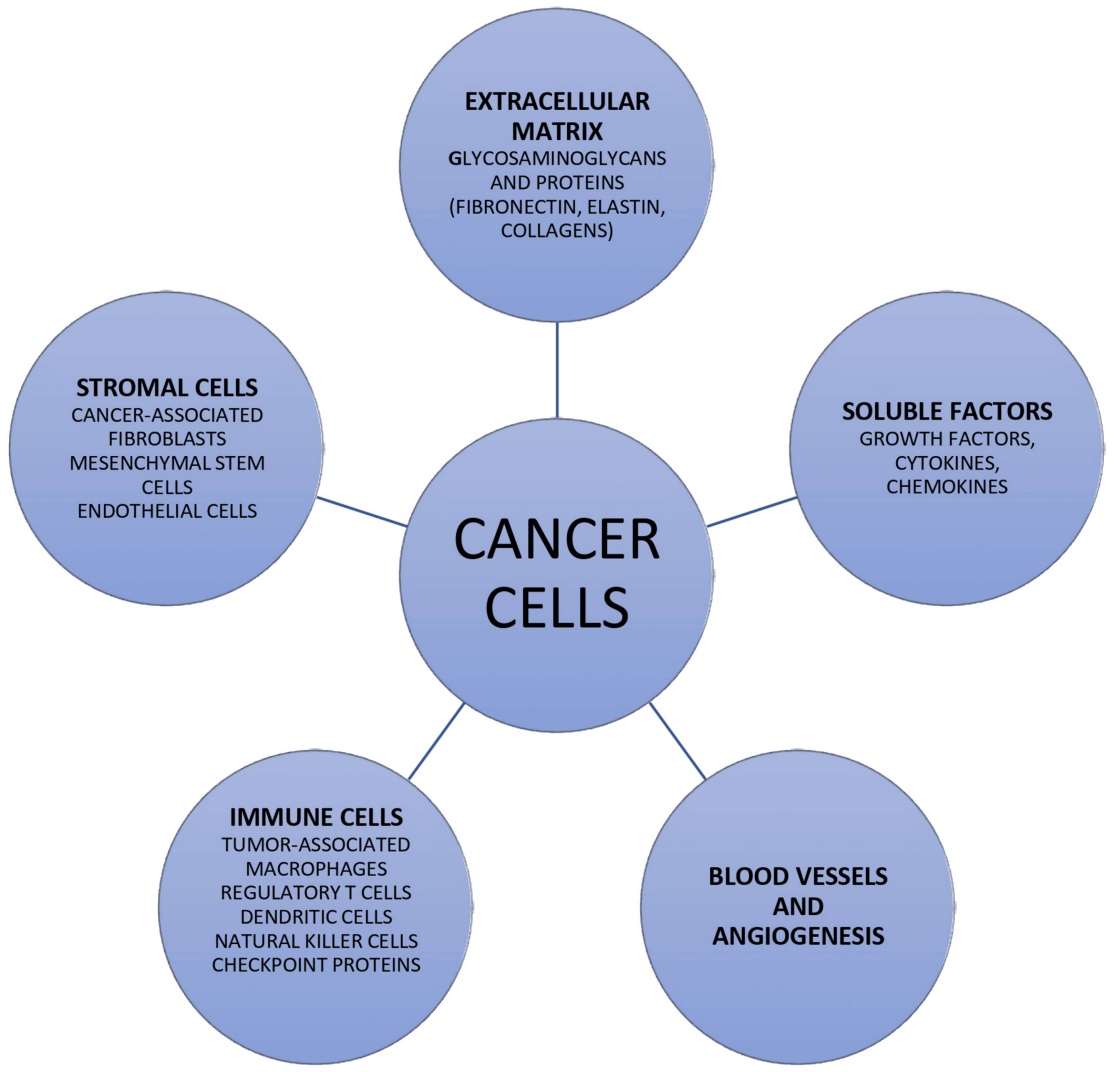
Components of TME TME: tumor microenvironment Image credit: Sohaila Fatima

Figure 1 shows the cancer cells and TME.

Stromal cells

Cancer-Associated Fibroblasts (CAFs)

The largest number of stromal cells in the TME are CAFs. They influence cancer cell invasion, migration, and resistance to treatment by secreting soluble molecules and extracellular matrix (ECM) proteins [27]. Mesenchymal stem cells (MSCs) can be drawn to tumors and aid in the formation of those tumors by differentiating into different cell types, such as CAFs, and by secreting growth hormones and cytokines that promote tumor growth [28]. Endothelial cells** **are vital to angiogenesis, which is necessary to provide oxygen and nourishment to tumors, allowing them to grow and spread [18].

Immune Cells

TAMs, or tumor-associated macrophages, are key immune cells that can play either pro- or anti-tumorigenic roles. TAMs take on an M2-like character in many cancers, which encourages angiogenesis, immunological suppression, and ECM remodeling [29]. Tregs, or regulatory T-cells, are implicated in the TME's suppression of the immunological response. By preventing effector immune cells from functioning, they aid tumors in avoiding immune monitoring and promote tumor development and metastasis [30]. Dendritic cells (DCs) are cells that display antigens and have the ability to elicit an immune response. Their capacity to stimulate T-cells, however, may be compromised in the TME, which could result in immunological evasion [31]. Natural killer (NK) cells are engaged in the surveillance and elimination of tumor cells. Nevertheless, they frequently undergo functional inhibition in the TME, which aids in tumor immune evasion [32].

Immunosuppressive nature: With a mix of immune checkpoint molecules (like programmed death-ligand 1 (PD-L1) with its receptor, programmed cell death protein 1 (PD-1), cytokines (like transforming growth factor-β (TGF-β) and interleukin-10 (IL-10), and immune cells (such Tregs and TAMs), the TME is usually immunosuppressive and inhibits efficient anti-tumor immune responses [33].

Tumor immunological evasion mechanisms: Tumors use a variety of strategies, including downregulating antigen presentation, activating immunological checkpoint pathways, and secreting immunosuppressive substances, to avoid immune detection [34].

Blood vessels and angiogenesis

Abnormalities in Tumor Blood Vessels

Both structural and functional abnormalities are frequently found in tumor blood vessels. They may have uneven blood flow, leakiness, and poor organization, all of which can lead to hypoxia in the TME and reduce the effectiveness of medication administration [18].

Angiogenesis's Function

Tumor growth and metastasis depend on angiogenesis, the creation of new blood vessels. Angiogenic factors, such as VEGF (vascular endothelial growth factor), are secreted by tumors and promote blood vessel expansion, providing a sufficient supply of oxygen and nutrients [35].

Consequences for Resistance and Drug Delivery

Treatment resistance is exacerbated by the tumor vasculature's disorganized structure, which makes it difficult to distribute chemotherapeutic drugs effectively. By encouraging the survival of cancer cells, hypoxic areas within tumors can result in resistance to specific treatments.

Extracellular matrix (ECM)

The extracellular matrix (ECM) is a complex network of glycosaminoglycans and structural proteins such as fibronectin, elastin, and collagens, which provides mechanical support and biochemical signaling to surrounding cells. In the TME, the ECM undergoes extensive remodeling, primarily driven by stromal cells, especially CAFs. These cells alter ECM composition, stiffness, and organization, creating a microenvironment that promotes tumor progression [36]. This remodeling process involves the increased deposition and activity of matrix metalloproteinases (MMPs), which degrade ECM components and facilitate cancer cell migration, invasion, and metastasis [37]. The resulting ECM becomes stiffer and more disordered, enhancing the ability of tumor cells to infiltrate nearby tissues and spread to distant organs. Given its critical role in cancer progression, the ECM is being explored as a therapeutic target. Strategies under investigation include inhibiting enzymes like MMPs that drive ECM remodeling and modulating ECM stiffness to reduce cancer cell invasiveness [38].

Soluble factors

Growth Factors, Cytokines, and Chemokines

Both tumor and stromal cells secrete growth factors (like vascular endothelial (VEGF) and epithelial growth factor (EGF), cytokines (like IL-6 and IL-10), and chemokines (like chemokine ligand 2 (CCL2) and C-X-C motif chemokine 12 (CXCL12). In the TME, they control immunological responses, migration, cell survival, and proliferation [39].

Impacts on Drug Resistance, Cell Survival, and Proliferation

Many of these soluble substances promote tumor survival by triggering signaling pathways that are beneficial to survival (e.g., the MAPK/ERK (mitogen-activated protein kinase/extracellular signal-regulated kinase signaling pathway) and PI3K/Akt (lipid kinase phoshoinositide-3-kinase signaling pathway). Through modifications to drug metabolism, decreased drug absorption, or improved anti-apoptotic pathways in cancer cells, they can also increase drug resistance [40].

TME in cancer development

Tumor Initiation and Early Development

An essential function of stromal cells is to establish a favorable environment for the development of tumors [6]. The TME can cause epigenetic changes and genetic alterations that reduce tumor suppressor genes or cause oncogene expression [16].

Tumor Progression

Cytokines, growth factors, and other signaling molecules secreted by stromal cells promote the survival and multiplication of cancer cells [6]. Cell adhesion, migration, and invasion are encouraged by the signaling communication and structural support that the ECM provides [6]. By interacting with the ECM through integrins, tumor cells can stimulate cell signaling pathways that enhance cell cycle progression and survival [41]. TAMs have the ability to generate pro-inflammatory cytokines that encourage angiogenesis and tumor cell development [42]. To promote tumor growth and progression, ECM is remodeled. Collagen, fibronectin, laminin, and other ECM proteins are produced by CAFs and other stromal cells and are broken down by MMPs [43]. ECM stiffness may encourage pro-tumorigenic signaling and increase treatment resistance [44]. By supplying resources and enhancing tumor cell survival in nutrient-deficient environments, stromal cells also have an impact on the metabolic microenvironment [45]. The TME attracts immune cells that can have pro-tumor effects, such as TAMs, Tregs, and MDSCs [46]. In order to mitigate anti-tumor immunity and encourage tissue remodeling, TAMs transform into M2 phenotype and release cytokines such as IL-10 and TGF-β [29]. Tregs aid the tumor in avoiding immune monitoring by inhibiting the function of cytotoxic T-cells [47].

Metastasis and Tumor Spread

In order to infiltrate local tissues and reach blood vessels, tumor cells need to engage with both stromal cells and the extracellular matrix [48]. The epithelial-to-mesenchymal transition (EMT), in which tumor cells lose their adhesive qualities and acquire the capacity to migrate and invade, is triggered by signals from the TME. Numerous TME constituents, such as growth factors, cytokines, and ECM remodeling enzymes, can produce these signals [49]. Proteases like MMPs aid in the breakdown of ECM components through ECM remodeling, which permits tumor cells to infiltrate the surrounding stroma [43]. Tumor cells extravasate after they enter the bloodstream, allowing them to spread to other tissues [50]. Tumor cells have the ability to precondition the TME in distant organs to serve as a supportive niche for metastatic growth; this process is referred to as the "pre-metastatic niche" [51]. Integrins allow tumor cells to interact with the extracellular matrix (ECM), and stromal cells can alter the ECM to make it more conducive to invasion [14]. New blood vessels produced by tumor-induced angiogenesis give tumor cells a way to enter the bloodstream (intravasation) and then spread out into other tissues. Because they provide easy access to the bloodstream, the aberrant and leaky blood arteries within the TME further encourage the dissemination of tumor cells [18].

Therapeutic strategies targeting the tumor microenvironment 

CAFs and Angiogenesis

CAFs are a promising therapeutic target because they remodel the extracellular matrix and secrete growth factors that support tumor progression [5]. Anti-angiogenic therapies, such as bevacizumab, aim to normalize tumor vasculature, thereby reducing the tumor’s access to oxygen and nutrients. However, these approaches are often limited by tumor resistance and adaptive mechanisms [52].

Immune Modulation

Reprogramming the TME to a tumor-suppressive state is another key strategy. Immunotherapies, particularly immune checkpoint inhibitors like anti-PD-1/PD-L1, have revolutionized cancer treatment by restoring T-cell functionality [53]. Additionally, therapies targeting TAMs aim to shift their phenotype from pro-tumorigenic (M2) to anti-tumorigenic (M1), thereby enhancing immune responses. Despite significant therapeutic breakthroughs, these strategies benefit only a subset of patients, highlighting the complexity of the TME [54,55].

Metabolic Reprogramming and Nanotechnology-Based Delivery Systems

Tumor and stromal cell metabolism is often altered, characterized by increased glycolysis (the Warburg effect) [56]. Targeting these metabolic pathways can disrupt communication between stromal and cancer cells, offering new therapeutic avenues [57].

Treatments Targeting Hypoxia

Improving tumor oxygenation and targeting hypoxia-related pathways, primarily through modulation of hypoxia-inducible factors (HIFs), can enhance the efficacy of cancer therapies [58].

Combining TME-targeted strategies with conventional treatments such as chemotherapy, radiotherapy, and immunotherapy holds promise for overcoming challenges like drug resistance, immune evasion, and limited treatment penetration. This integrative approach may lead to more effective cancer therapies [59].

Table 1 summarizes current therapeutics targeting the TME.

**Table 1:** Population data table for drugs evaluated/being evaluated for targeting TME CSF-1: colony stimulating factor 1, CSF-1R: colony stimulating factor 1 receptor, FAP-α: fibroblast activation protein alpha, IL-6: interleukin 6, IL-10: interleukin 10, TGF-β: transforming growth factor β, siRNA: silencing RNA, VEGF: vascular endothelial growth factor, TME: tumor micorenvironment

Target	Therapeutic strategy	Example/approach
Tumor-associated macrophages (TAMs)	Depletion, reprogramming	CSF-1/CSF-1R inhibitors (e.g., PLX-3397) [60]
Cancer-associated fibroblasts (CAFs)	Targeting specific markers	Cleavable amphiphilic peptide nanoparticles (CAP-NPs) responsive to FAP-α [61]
Myeloid-derived suppressor cells (MDSCs)	Blocking immunosuppressive activity	Phosphodiesterase-5 inhibitors, cyclooxygenase-2 inhibitors [60]
T-cells	Enhancing activity	Immune checkpoint inhibitors (e.g., pembrolizumab, nivolumab) [62]
Cytokines	Modulation	Adjusting levels of IL-6, IL-10, and TGF-β [62]
Extracellular vesicles (EVs)	Drug delivery	Using EVs to deliver siRNA and medications to tumor cells [62]
Hypoxia	Nanoparticle activation	Oxygen-sensitive nanoparticles [60]
Acidic pH	Nanoparticle activation	pH-responsive nanoparticles [60]
Angiogenesis	Inhibition	Anti-VEGF therapy [61]
Metabolic pathways	Disruption	Targeting glycolysis and oxidative phosphorylation [62]

Targeted agents approved for cancer treatment

Targeted therapies have completely changed the therapeutic landscape by providing more accurate and frequently less harmful substitutes for conventional chemotherapy in the treatment of cancer [63]. Here, we review various targeted medicines, emphasizing their potential in distinct cancer types, clinical significance, and mechanisms of action.

Tyrosine Kinase Inhibitors

An innovative oral multitargeted tyrosine kinase inhibitor (sunitinib malate) with anticancer and antiangiogenic properties. Recently, it has been approved as a first-line treatment for patients with advanced renal cell carcinoma (RCC) and gastrointestinal stromal tumors (GIST) in cases where imatinib mesylate therapy was ineffective or the patient's disease progressed [64].

Sorafenib

The multikinase inhibitor is approved for the treatment of advanced renal cell carcinoma (RCC). It dramatically increases survival for individuals with advanced HCC, according to a recent phase III trial [65]. It inhibits a number of kinases like Raf, VEGFR, and PDGFR that are involved in angiogenesis and tumor cell proliferation [66].

PD-1 and PD-L1 Inhibitors

T-cells contain the checkpoint protein PD-1 receptors [67]. It inhibits their immunological response when it binds to antigen-presenting cell (APC) ligands PD-L1 and PD-L2. The regulation of peripheral and central tolerance depends heavily on this system [68]. PD-L1 is abundant in certain cancer cells, which aids in their ability to evade an immune response.

PD-1 blockers: PD-1-targeting medications include cemiplimab, nivolumab, and pembrolizumab [67].

Inhibitors of PD-L1: Drugs that target PD-L1 include durvalumab, atezolizumab, and avelumab [67].

Nivolumab

A human monoclonal IgG4 antibody called nivolumab has a high affinity and specificity for binding to PD-1 receptors. The FDA currently approves nivolumab for the treatment of colorectal cancer with microsatellite instability, hepatocellular carcinoma, classical Hodgkin lymphoma, esophageal squamous cell carcinoma, pleural mesothelioma, small cell lung cancer, urothelial carcinoma, melanoma, renal cell carcinoma, non-small cell lung cancer, and squamous cell carcinoma of the head and neck [69].

Angiogenesis Inhibitors

These block the formation of new blood vessels that feed and nourish the cancer cells. Examples include monoclonal antibodies targeting vascular endothelial growth factor (VEGF) (bevacizumab, ramucirumab, olaratumab); recombinant fusion proteins (aflibercept); mammalian target of rapamycin (mTOR) inhibitors (temsirolimus, everolimus); immunomodulatory agents (thalidomide, lenalidomide); tyrosine kinase inhibitors (sorafenib, sunitinib, pazopanib, vandetanib, regorafenib, axitinib, ponatinib, cabozantinib, apatinib, nintedanib, lenvatinib) [70].

CAR T-cell Therapies

Chimeric antigen receptor T-cell therapy is a form of adoptive T-cell immunotherapy (ACT). ACT involves using a patient’s own immune cells, particularly T-cells, and enhancing their ability to recognize and attack cancer cells. These engineered T-cells are then infused back into the patient to target and eliminate cancer cells more effectively [71]. Examples of CAR T-cell therapies currently approved include tisagenlecleucel, axicabtagene ciloleucel, brexucabtagene autoleucel, lisocabtagene maraleucel, idecabtagene vicleucel, ciltacabtagene autoleucel, and obecabtagene autoleucel [67].

Cytotoxic T Lymphocyte-Associated Antigen-4 (CTLA-4) Inhibitors

After T-cell activation, CTLA-4 is displaced to the plasma membrane and functions as a T-cell activation inhibitor. Anti-CTLA-4 antibody binds with CTLA-4, which results in T-cell reactivation. Examples of drugs are ipilimumab and tremelimumab [72]. 

LAG-3 Inhibitors

LAG-3 (CD223) has a regulatory role that consists of the inhibition of immune function, cell proliferation, homeostasis, and cytokine secretion [73].

Cytokines

Interleukin-2 (IL-2) helps immune system cells grow and divide more quickly. A man-made version of IL-2 is approved to treat advanced renal cancer and metastatic melanoma. IFN-alfa can be used to treat cancers such as hairy cell leukemia, chronic myelogenous leukemia (CML), follicular non-Hodgkin lymphoma, cutaneous (skin) T-cell lymphoma, kidney cancer, melanoma, and Kaposi sarcoma [67].

Limitations of TME for cancer treatment

The TME presents significant challenges for cancer treatment due to its complex and dynamic nature. Hypoxia, a hallmark of the TME, promotes genetic instability and angiogenesis, leading to the formation of abnormal blood vessels [74]. These vascular abnormalities result in poor perfusion and irregular blood flow, hindering drug delivery and creating areas of high interstitial fluid pressure [75]. TME and its immunosuppressive components can influence both tumor progression and response to treatment. The cytokine-mediated recruitment of various suppressive cells into the TME rapidly becomes a barrier to therapy as the accumulation of normal cells can occupy a large fraction of the tumor mass. When these normal cells promote tumor growth rather than suppress it, the situation is problematic [76]. Stromal cell interactions, particularly with cancer-associated fibroblasts, contribute to treatment resistance by secreting various factors that support tumor growth and survival [33]. Metabolic alterations within the TME create an environment that enhances therapy resistance and tumor progression. The acidic nature of the TME further impairs drug effectiveness and promotes tumor progression [77].

The inherent heterogeneity of the TME, coupled with the intricate interactions between tumor cells, stromal components, and the immune system, poses substantial challenges in both basic research and therapeutic development. These challenges are further compounded in preclinical modeling, where replicating the complexity, spatial organization, and dynamic evolution of the TME remains a significant hurdle [78]. Conventional in vitro systems often fail to capture the full spectrum of TME interactions, while in vivo models may not fully recapitulate human-specific immune and stromal responses. Overcoming these barriers requires not only an enhanced understanding of the molecular and cellular components of the TME but also the development of more sophisticated and physiologically relevant models (such as 3D organoids, organ-on-chip systems, and humanized mouse models) that more accurately represent its diversity and dynamic nature [79]. Advancing these areas of research will be critical in devising more effective, personalized, and targeted treatments for cancer patients, ultimately improving clinical outcomes [80].

## Conclusions

Future directions in TME research and cancer treatment are focused on addressing the complex challenges presented by the TME and leveraging new technologies to improve therapeutic outcomes. One promising avenue is the development of combination therapies that target both tumor cells and specific components of the TME. This approach aims to normalize tumor vasculature, improve drug delivery, and create a more favorable environment for immune cell function. Targeting specialized microenvironments within the TME, such as hypoxic niches and immune microenvironments, is another area of focus. Understanding the crosstalk between these compartments could lead to more effective treatment strategies.

Emerging technologies like cold plasma therapy show potential in modulating cell-to-cell and cell-to-ECM communication within the TME, potentially propagating therapeutic effects beyond the local treatment area. Exploring the role of extracellular pH in the TME and its impact on immunotherapy efficacy represents a new frontier in cancer research. These advancements collectively aim to overcome the limitations of current treatments and improve patient outcomes. Artificial intelligence (AI) is emerging as a powerful tool for analyzing the intricate TME. By leveraging AI to process vast amounts of data from multiple sources, researchers can uncover novel insights into TME dynamics, predict treatment responses, and guide more personalized cancer therapies.
